# Association of metabolic syndrome with various anthropometric and atherogenic parameters in the Kazakh population in China

**DOI:** 10.1186/s12944-016-0338-9

**Published:** 2016-09-23

**Authors:** Xiaocui Chen, Chunhui He, Yitong Ma, Yining Yang, Fen Liu, Xiang Ma, Xiaomei Li, Xiang Xie, Bangdang Chen

**Affiliations:** 1 Xinjiang Key Laboratory of Cardiovascular Disease, Clinical Medical Research Institute, First Affiliated Hospital of Xinjiang Medical University, Urumqi, 830054 China; 2 Department of Cardiology, First Affiliated Hospital of Xinjiang Medical University, No. 137 South Liyushan Road, Urumqi 830054 People’s Republic of China

**Keywords:** Metabolic syndrome, Body mass index, Waist to height ratio, Waist to hip ratio, Body adiposity index, TG/HDL-C

## Abstract

**Background:**

This study aimed to evaluate the association of the metabolic syndrome (MetS) with various anthropometric and atherogenic parameters in adult Kazakh population in China.

**Methods:**

Four thousand ninety-four Kazakhs were recruited since 2007 to 2010. MetS and its components were confirmed according to IDF criteria. Area under the curve (AUC) of each variable was compared. Sensitivity (Sen), specificity (Spe), shortest distance in receiver’s operating characteristic curve (ROC) and cutoff of each variable to diagnose MetS were calculated.

**Results:**

28.6 % of men and 31.0 % of women had MetS in the Kazakh population. In men, WHtR had the highest AUC value 0.821, followed by BMI (0.801), TG/HDL-C (0.792), WHR (0.776) and BAI (0.666). In women, WHtR also had the highest AUC value (0.835), following by BMI (0.789), WHR (0.778), TG/HDL-C (0.778) and BAI (0.751). WHtR had the shortest ROC distance that was 0.37 and the optimal cutoff was 0.55 in men. In women, WHtR also had the shortest ROC distance of 0.35 and the optimal cutoff was 0.54.

**Conclusion:**

WHtR is the best predictor of MetS in both Kazakh men and women according to the IDF criteria.

## Background

Metabolic syndrome (MetS) is a cluster of metabolic abnormalities characterized as central obesity, raised triglyceride (TG) level, reduced high density lipoprotein cholesterol (HDL-C), raised blood pressure, raised fasting plasma glucose, or previously diagnosed type 2 diabetes [1]. It will exacerbate the progression of cardiovascular disease (CVD) if left untreated [2]. In recent years, many studies have focused on optimal obese and atherogenic indices to predict metabolic syndrome [3–5]. Nevertheless, controversy still remains over which anthropometric parameters convey the highest risk of metabolic syndrome.

Body mass index (BMI) has been used to assess body fat for almost 200 years. Waist to height ratio (WHtR) has recently been regarded as the best screening tool for detection of cardiometabolic risk factors, especially in Asian populations [6–9]. Some studies proposed waist circumference (WC) or waist to hip ratio (WHR) [10–12] as the best anthropometric parameters to predict the metabolic syndrome, whereas others advocated their combined use [13, 14]. Although BMI, WHtR, WC and WHR are simple and convenient anthropometric parameters for epidemiological studies, their validity in measuring adiposity has been questioned since they cannot differentiate between fat and lean mass [15]. One study had reported that body adiposity index (BAI) could be used to reflect %body fat for adult men and women of different ethnicities without numerical correction [3]. A study based on Chinese postmenopausal women showed that TG/HDL-C is the best to predict the presence of metabolic syndrome [5].

This cross-sectional study aimed to identify the association of metabolic syndrome with various anthropometric and atherogenic parameters (BMI, WHtR, WHR, BAI and TG/HDL-C) in Kazakh population in Xinjiang Uighur Autonomous Region, northwest of China.

## Results

### Baseline characteristics of the study participants

Table 1 summarized the baseline characteristics of the participants with or without MetS in Kazakh men and women. In men, the participants with MetS tended to have significantly elder age and higher height, weight, waist circumference, hip circumference, SBP, DBP, fasting glucose, total cholesterol, triglycerides, BMI, WHR, WHtR, BAI, TG/HDL-C when compared with the participants without MetS (*P* < 0.001). The participants without MetS tended to have significantly higher HDL-C and LDL-C compared with the participants with MetS (*P* < 0.001). In women, the parameters were higher in participants with MetS than those in participants without MetS except for that the height, HDL-C and LDL-C were lower in participants with MetS compared with that in participants without MetS.

**Table 1:** Characteristics of the participants with or without MetS in Kazakh men and women

	Men (*n* = 1999)			Women (*n* = 2095)		
	With MetS (*n* = 571)	Without MetS (*n* = 1428)	*P* value	With MetS (*n* = 649)	Without MetS (*n* = 1446)	*P* value
Age (year)	50.61 ± 11.67	48.70 ± 12.14	0.001*	53.54 ± 10.20	45.57 ± 10.92	<0.001*
Waist circumference (cm)	101.77 ± 8.91	87.53 ± 11.26	<0.001*	95.27 ± 9.91	80.21 ± 12.02	<0.001*
Hip circumference (cm)	105.03 ± 7.77	97.98 ± 8.57	<0.001*	104.61 ± 10.00	96.40 ± 9.20	<0.001*
SBP (mmHg)	152.56 ± 22.87	137.37 ± 22.51	<0.001*	154.57 ± 25.01	131.62 ± 23.69	<0.001*
DBP (mmHg)	97.93 ± 17.85	87.18 ± 18.20	<0.001*	96.87 ± 20.27	81.52 ± 18.49	<0.001*
Fasting glucose (mmol/L)	5.91 ± 2.26	4.94 ± 1.05	<0.001*	5.56 ± 2.00	4.80 ± 0.97	<0.001*
Total cholesterol (mmol/L)	5.19 ± 1.25	4.65 ± 1.15	<0.001*	5.18 ± 1.09	4.54 ± 1.08	<0.001*
LDL-C (mmol/L)	2.77 ± 0.87	2.94 ± 0.93	<0.001*	2.78 ± 0.92	2.98 ± 0.94	<0.001*
HDL-C (mmol/L)	1.10 ± 0.41	1.36 ± 0.43	<0.001*	1.10 ± 0.34	1.38 ± 0.41	<0.001*
Triglycerides (mmol/L)	1.95 ± 1.55	1.08 ± 0.78	<0.001*	1.45 ± 0.82	0.92 ± 0.45	<0.001*
BMI (kg/m^2^)	29.99 ± 3.69	25.71 ± 3.79	<0.001*	29.68 ± 4.86	24.60 ± 4.50	<0.001*
WHR	0.97 ± 0.07	0.89 ± 0.09	<0.001*	0.91 ± 0.08	0.83 ± 0.08	<0.001*
WHtR	0.60 ± 0.05	0.52 ± 0.07	<0.001*	0.61 ± 0.07	0.51 ± 0.08	<0.001*
BAI	29.45 ± 4.11	27.14 ± 4.22	<0.001*	35.40 ± 5.71	30.88 ± 4.88	<0.001*
TG/HDL-C	1.99 ± 2.32	0.92 ± 1.13	<0.001*	1.45 ± 0.99	0.77 ± 0.65	<0.001*

*MetS* metabolic syndrome, *SBP* systolic blood pressure, *DBP* diastolic blood pressure, *LDL-C* low-density lipoprotein cholesterol, *TG* triglycerides, *HDL-C* high-density lipoprotein cholesterol, *BMI* body mass index, *WHR* waist to hip ratio, *WHtR* waist to height ratio, *BAI* body adiposity index

^*^ *P* value < 0.05 compared to With MetS

### Prevalence of MetS and its components

**Fig. 1:**
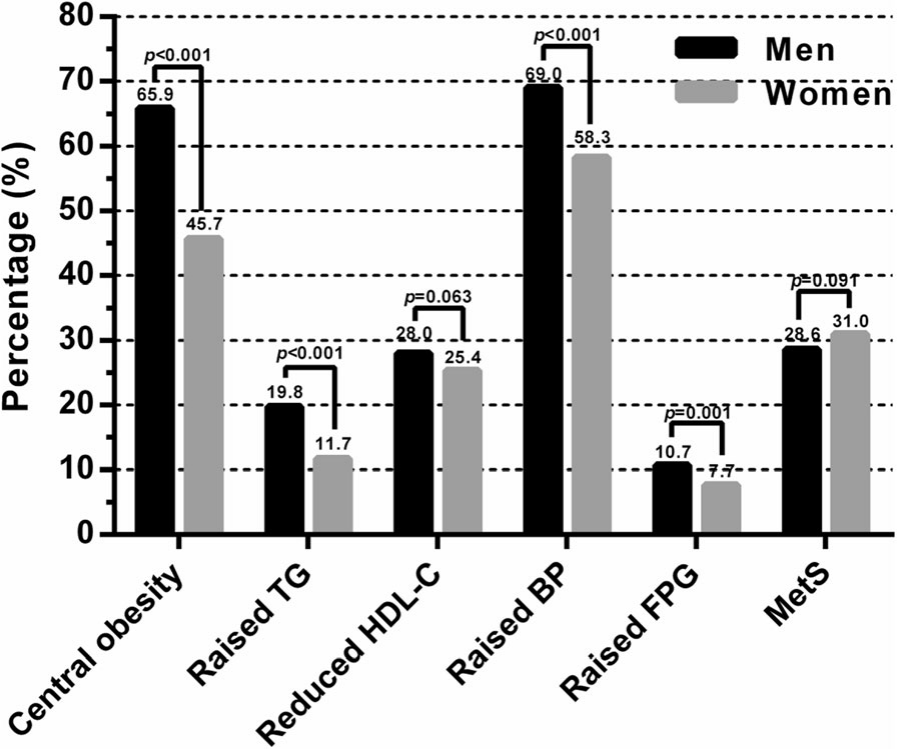
Age adjusted prevalence of MetS and its individual components in Kazakh men and women. MetS, metabolic syndrome; TG, triglycerides; HDL-C, high-density lipoprotein cholesterol; BP, blood pressure; FPG, fasting plasma glucose

Prevalence of MetS and its individual components in Kazakh men and women were shown in Fig. 1. After age adjustment, 28.6 % of men had the MetS while 31.0 % of women had the MetS, the prevalence of MetS in women and men were not significantly different (*P =* 0.091). As for the individual components of MetS, the prevalence of central obesity, raised TG, raised BP and raised FPG in women was significantly lower than that in men (*P* < 0.001). However, the prevalence of reduced HDL-C was not significantly different among men and women (*P* = 0.063).

### Association of each anthropometric and atherogenic parameter with MetS by ROC

**Fig. 2:**
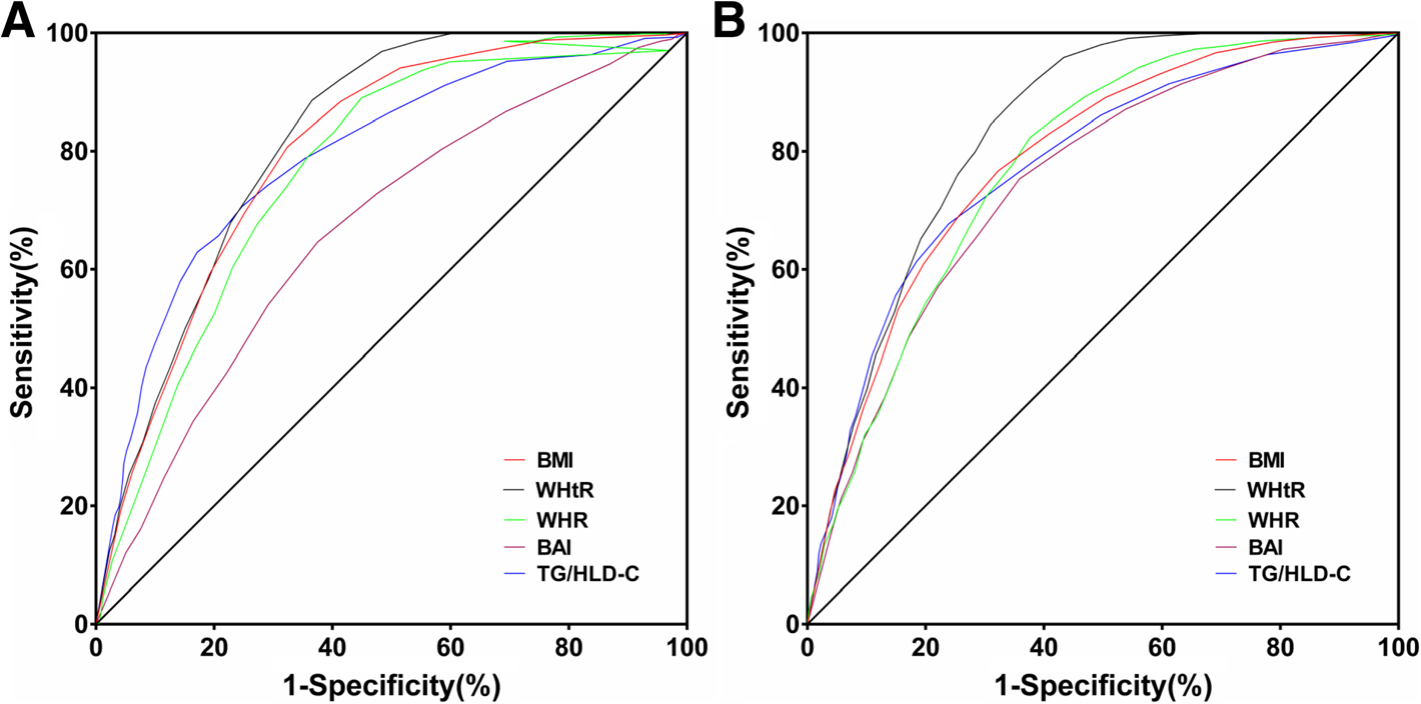
ROC curves for each variable to predict the presence of MetS in Kazakh men (**a**) and women (**b**). ROC, receiver operating characteristic; MetS, metabolic syndrome; BMI, body mass index; WHtR, waist to height ratio; WHR, waist to hip ratio; BAI, body adiposity index; TG, triglycerides; HDL-C, high-density lipoprotein cholesterol

Table 2 presented the AUC of each variable for the presence of MetS in Kazakh men and women. In men, according to the respective ROC curves, the WHtR had the highest AUC value (AUC = 0.821), it was followed by BMI (AUC = 0.801), TG/HDL-C (AUC = 0.792), WHR (AUC = 0.776) and BAI (AUC = 0.666). In women, according to the respective ROC curves, the WHtR also had the highest AUC value (AUC = 0.835), it was followed by BMI (AUC = 0.789), WHR (AUC = 0.778), TG/HDL-C (AUC = 0.778) and BAI (AUC = 0.751). Figure 2 showed ROC curves for each variable to predict the presence of MetS in Kazakh men and women.

**Table 2:** Area under the curves of each variable for the presence of MetS in Kazakh men and women

Varibles	AUC (95 % CI) in Men	AUC (95 % CI) in Women
BMI	0.801 (0.781, 0.821)	0.789 (0.769, 0.809)
WHR	0.776 (0.755, 0.796)	0.778 (0.758, 0.798)
WHtR	**0.821 (0.803, 0.839)**	**0.835 (0.818, 0.852)**
BAI	0.666 (0.640, 0.692)	0.751 (0.728, 0.773)
TG/HDL-C	0.792 (0.769, 0.815)	0.778 (0.756, 0.800)

*MetS* metabolic syndrome, *AUC* Area under the curves, *BMI* body mass index, *WHR* waist to hip ratio, *WHtR* waist to height ratio, *BAI* body adiposity index, *TG* triglycerides, *HDL-C* high-density lipoprotein cholesterol

The bolded data represent the highest AUC (areas under the curve) of ROC curve with the WHtR

### Cutoff of each anthropometric and atherogenic parameter in MetS with ROC

Table 3 showed the sensitivity, specificity, distance in the ROC and cutoffs of each variable for the presence of MetS in Kazakh men and women. In men, among all five anthropometric and atherogenic parameters, WHtR had the shortest distance of 0.37 in ROC (Sen = 81.09 %, Spe = 68.50 %), the cutoff of WHtR was 0.55. Following WHtR, the association of each parameter to MetS was followed by BMI, TG/HDL-C, WHR and BAI according to the distance in ROC. In women, among all five anthropometric and atherogenic parameters, WHtR also had the shortest distance of 0.35 in ROC (Sen = 84.59 %, Spe = 68.97 %), the cutoff of WHtR was 0.54. Following WHtR, the association was followed by BMI, TG/HDL-C, WHR, and BAI according to the distance in ROC.

**Table 3:** Sensitivity (Sen), specificity (Spe), distance in the receiver operating characteristic (ROC) curve and cutoffs of each variable for the presence of MetS in Kazakh men and women

	Anthropometric index	Sen (%)	Spe (%)	Distance in ROC curve	Cutoff
Men	BMI	80.74	67.60	0.38	27.0
	WHR	73.91	67.78	0.41	0.93
	**WHtR**	**81.09**	**68.50**	**0.37**	**0.55**
	BAI	64.62	62.49	0.52	28.00
	TG/HDL-C	70.66	75.25	0.38	1.10
Women	BMI	76.73	67.72	0.40	26.0
	WHR	72.42	69.71	0.41	0.87
	**WHtR**	**84.59**	**68.97**	**0.35**	**0.54**
	BAI	75.35	64.14	0.44	32.00
	TG/HDL-C	67.73	76.10	0.40	0.90

*MetS* metabolic syndrome, *AUC* Area under the curves, *BMI* body mass index, *WHR* waist to hip ratio, *WHtR* waist to height ratio, *BAI* body adiposity index, *TG* triglycerides, *HDL-C* high-density lipoprotein cholesterol

The boled part means the optimal value in respect sex

## Discussion

The total Kazakh population was 1.46 million in 2010 in China, 99.57 % of the total Kazakh population live in Xinjiang Uighur Autonomous Region that is located in the center of Asia. This study provides the population-based data on the prevalence of MetS in Kazakh population aged over 35 years. To our knowledge, this is the first large scale, population based, cross-sectional survey to estimate the prevalence of MetS in Kazakh population in Xinjiang, China. In this present study, 28.6 % of the men had MetS while 31.0 % of the women had MetS according to IDF criteria. The prevalence of MetS between men and women was not significantly different (*P* = 0.091), which was different from other studies both in China and other countries [16–18].

Age-standardized prevalence of MetS was 28.8 % according to IDF criteria based on Chinese postmenopausal women in Guangdong province [5]. The age-standardized prevalence of MetS was 28.6 % in women based on elderly Chinese adults in Jiangsu province [19]. Our study investigated the Kazakh population within the same age range. We found that the prevalence of MetS in Kazakh women was higher than that in Chinese women in Guangdong province [4, 5]. One study including a total of 15,838 Chinese individuals showed that 31.1 % of female participants had BMI ≥ 25, 37.8 % of female participants had the WC > 80 cm [20]. The reason for the higher prevalence of MetS and obesity in Kazakh women was still unclear. The eating and living habit may play an important role, the Kazakh population consumed more pasta, meat and milk products than the Han population did. The majority of Kazakh women tended to consume food containing a large amount of calories and lipids for a long time after they gave birth, it may greatly contribute to the high prevalence of MetS and obesity in Kazakh women.

In men, the WHtR had the highest AUC value of 0.821, it was followed by BMI, TG/HDL-C, WHR and BAI. Similarly, among all five anthropometric and atherogenic parameters, WHtR had the shortest distance of 0.37 in ROC. The association of WHtR to MetS was followed by BMI, TG/HDL-C, WHR and BAI according to the distance in ROC. Our results were consist with the study by Zhang et al. [4], they reported WHtR was best predicting indicator of MetS in Chinese men in Gunagdong province with the AUC of 0.69. The reason why WHtR may be a better predictor of metabolic risk was that it took height into account. An association between short height and risk of coronary heart disease was previously reported [21]. Henriksson et al. found that body height was negatively related to serum cholesterol and non-HDL cholesterol in middle-aged men, which was independent of BMI and WHR [22]. Thus, it may be important to take height into consideration. They also reported that WC had the equivalent predicting performance as the WHtR since the AUC was also 0.69. In this present study, WC was the necessary factor in the diagnosis of MetS according to IDF criteria [23], so the AUC of WC was 100 %. The AUC value of WHtR in our study was 0.821, which was larger than 0.69 in study mentioned above. It suggested that WHtR may be a better indicator for MetS especially in Kazakh men, the sensitivity was 81.09 % and specificity was 68.50 %.

In women, according to the respective ROC curves, the WHtR had the highest AUC value of 0.835, it was followed by BMI, WHR, TG/HDL-C and BAI. Meanwhile, WHtR had the shortest distance of 0.35 in ROC (Sen = 84.59 %, Spe = 68.97 %). The association of WHtR to MetS was followed by BMI, TG/HDL-C, WHR, and BAI according to the distance in ROC. Our results were consistent with the study by Zhang et al. [4], they reported the WHtR was best predicting indicator of MetS in Chinese women with the AUC of 0.70. Therefore, this atherogenic index may be used as the best parameter in the screening of MetS in Chinese women. Even BAI was reported to be able to reflect %body fat of differing ethnicities without numerical correction, however, the predicting ability of BAI was the lowest in both Kazakh men and women in our study.

The cut-off values of the parameters to detect the MetS in Kazakh men were 27.00 kg/m^2^, 0.93, 0.55, 28.00, 1.10 for BMI, WHR, WHtR, BAI, TG/HDL, respectively. Meanwhile, the cut-off values of the parameters to detect the MetS in Kazakh women were 26.00 kg/m^2^, 0.87, 0.54, 32.00, 0.90 for BMI, WHR, WHtR, BAI, TG/HDL, respectively. BMI and WHtR values in both man and women of our study were higher than those in Japanese and Korean man and women [24, 25]. We also noticed that cut-off values of BMI, WHR, WHtR and TG/HDL in Kazakh women were also higher than those in Chinese women in Guangdong [5]. The reason why the cutoffs to detect the MetS in Kazakh population were at high levels was still unclear. The ethnic difference and living habits may greatly contribute to it.

The present study has several strengths. This is the first representative sample of the general adult Kazakh population in China. Thus, these results can be generalized to the full adult Kazakh population aged above 35 years. Additionally, we provided AUC and cutoffs information for many anthropometric and atherogenic parameters stratified by sex. Future studies can use the cutoffs suggested here for the screening and intervention of MetS in a representative sample of the adult Kazakh population. The limitation of this study is its cross-sectional design, which precludes causal inferences. Second, CRS was conducted based on one time visit, the prevalence of raised blood pressure and raised fasting plasma glucose may be underestimated to some extent.

## Conclusion

In this study of adult Kazakh population showed that 28.6 % of men had the MetS, while 31.0 % of women had the MetS in Xinjiang, China. Among all five anthropometric and atherogenic parameters, the WHtR had closest association with MetS both in men and women. This study enriched the limited data on the MetS for immediate use in screening among Kazakh population.

## Methods

### Ethics statement

This study was approved by the Ethics Committee of The First Affiliated Hospital of Xinjiang Medical University and was conducted according to the standards of the Declaration of Helsinki. Written, informed consent was obtained from the participants [26].

### Study population

All the participants were selected from the Cardiovascular Risk Survey (CRS) study, the detailed description of the study population and the methods were described previously [27, 28]. Briefly, the CRS study used a 4-stage stratified sampling method to select a representative sample of the general population in Xinjiang, northwest of China. The research sites included Urumqi City, Kelamayi City, Fukang City, Yili Prefecture. The time period was from Oct. 2007 to Mar. 2010. The selections made from sampling units were based on geographic area, sex, and age groups using household registries. The 4-stage stratified sampling method was as follows: Stage one, according to population census data of Xinjiang in 2000, the areas mentioned above were selected based on population, ethnicity, geography, economic and cultural development level respectively. Stage two, according to the ethnic aggregation status, one district or county was randomly selected from the Kazakh population dominated area. Stage three, one community or town (village) was randomly selected from each district or county. Stage four, subjects over 35 years were randomly selected from each community or town (village) as research subjects. The staff conducted surveys in households and administered questionnaires. The questionnaires included the demographic, socioeconomic, dietary, and medical history of each participant. In total, the CRS included 14,618 participants (5757 Hans, 4767 Uighurs, and 4094 Kazakhs). Four hundred ninety-four Kazakh participants were enrolled in the present study. One thousand nine hundred ninety-nine participants were male and 2095 participants were female. The age of the participants were from 35 to 98 years old with the mean ± SD age of 48.63 ± 11.68 (men, 49.25 ± 12.03; women, 48.03 ± 11.31).

### Data collection

Data collection involved one visit in the participants’ residential areas. During the examinations, a standard questionnaire assessing demographic information and medical history was collected by trained research staff. Body weight was measured while the subjects were without clothes or shoes by using a double balance placed on a firm surface. Height was measured by using a Frankfort place positioned at a 90° angle against a wall-mounted metal tape. Waist and hip circumferences were measured at the level of the umbilicus, and at the level of the maximum girth between the iliac crest and the crotch, respectively. BMI was calculated as weight in kg divided by height in m^2^. WHR was calculated as the ratio of the waist-to-hip circumferences. WHtR was calculated by dividing waist circumference by height. Body adiposity index was calculated using the following equation: BAI = ((hip circumference)/((height)^1.5^) − 18) [3].

### Laboratory methods

Blood samples were obtained from the antecubital vein into anticoagulant tubes containing EDTA in the morning after an overnight fasting period. All the collected samples were transported on dry ice at prearranged intervals to Xinjiang Coronary Artery Disease VIP Laboratory. The serum concentration of serum total cholesterol (TC), triglyceride (TG), low density lipoprotein (LDL), high density lipoprotein (HDL) and fasting glucose were measured by the Clinical Laboratory Department of The First Affiliated Hospital of Xinjiang Medical University with the biochemical analyzer (Dimension AR/AVL Clinical Chemistry System, Newark, NJ, USA) [29, 30].

### Blood pressure measurement

A mercury sphygmomanometer was used to measure the blood pressure in the sitting position after a 10 min rest period. During the 30 min preceding the measurement, the subjects were required to refrain from smoking or consuming caffeine. The appearance of the first sound was used to define systolic blood pressure, and the disappearance of sound was used to define diastolic blood pressure [31]. Systolic and diastolic blood pressures were measured for twice, and the average of measurements was used for data analysis. If the first two measurements differed by more than 5 mmHg, additional readings were taken.

### Definition of metabolic syndrome

Following the IDF criteria, metabolic syndrome was defined by central obesity (defined as waist circumference ≥ 90 cm for Chinese men and ≥ 80 cm for Chinese women, with ethnicity specific values for other groups), plus any two of the following four factors: Raised TG level: ≥ 150 mg/dL (1.7 mmol/L), or specific treatment for this lipid abnormality; Reduced HDL cholesterol: < 40 mg/dL (1.0 mmol/L) in males and < 50 mg/dL (1.3 mmol/L) in females, or specific treatment for this lipid abnormality; Raised blood pressure: systolic BP ≥ 130 or diastolic BP ≥ 85 mmHg, or treatment of previously diagnosed hypertension; Raised fasting plasma glucose (FPG) ≥ 100 mg/dL (5.6 mmol/L), or previously diagnosed type 2 diabetes; If FPG was above 5.6 mmol/L or 100 mg/dL, OGTT is strongly recommended but is not necessary to define presence of the syndrome [23].

### Statistics

The statistical analysis was conducted using SPSS version 17.0 for Windows (SPSS Inc., Chicago, IL, USA). Continuous variables were expressed as gender and metabolic syndrome specific means and standard deviations, and categorical variables were expressed as gender-specific proportions. Age-standardization was performed by the direct method by using the 2000 Xinjiang Kazakh population as the standard population. The area under the receiver’s operating characteristic curve (AUC) and the 95 % confidence intervals (CIs) were used to compare the association of various anthropometric and atherogenic parameters. Additionally, the distance on the receiver operating characteristic (ROC) curve was calculated as the square root of [(1 - sensitivity)^2^ + (1 - specificity)^2^]. The parameter with the shortest distance on the ROC curve, which means the minimum distance from ROC curve to the upper left corner, was considered as the appropriate cutoff.

## Abbreviations

BAI: Body adiposity index
BMI: Body mass index
DBP: Diastolic blood pressure
HDL: High-density lipoprotein cholesterol
LDL-C: Low-density lipoprotein cholesterol
MetS: Metabolic syndrome
SBP: Systolic blood pressure
TG: Triglycerides
WHR: Waist to hip ratio
WHtR: Waist to height ratio
